# Application of Rupantaran software to Sri Lankan hospitals: an innovative tool developed to merge population-based cancer registry data into CanReg5

**DOI:** 10.3332/ecancer.2023.1553

**Published:** 2023-05-25

**Authors:** Pratik Sawant, Suraj Perera, Kelaniyan Godage Nirmala Jayanthi, Ahamed Ismail Ahamed Ziyad, Sushama Saoba, Morten Ervik, Leslie Mery, Freddie Bray, Rajesh Dikshit, Atul Budukh

**Affiliations:** 1 Centre for Cancer Epidemiology, Tata Memorial Centre, Kharghar, Navi Mumbai 410210, Maharashtra, India; 2 Strategic Information Management Unit, National Cancer Control Programme, Colombo 00500, Sri Lanka; 3 Cancer Surveillance Branch, International Agency for Research on Cancer, 69366 Lyon Cedex 07, France; 4 Homi Bhabha National Institute, Training School Complex, Anushakti Nagar, Mumbai 400094, India; a https://orcid.org/0000-0002-8781-4618; b https://orcid.org/0000-0002-6842-0330; c https://orcid.org/0000-0003-4485-3577; d https://orcid.org/0000-0002-3248-7787; e https://orcid.org/0000-0003-4830-0486; f https://orcid.org/0000-0001-6723-802X

**Keywords:** population-based cancer registry, cancer control, CanReg5 software, India, Sri Lanka

## Abstract

The purpose of a population-based cancer registry is to provide information on the disease burden for cancer control planning and is essential in studies on assessing the effectiveness of prevention, early detection, screening and cancer care interventions, where implemented. Sri Lanka is one of the Member States of the World Health Organisation’s South-East Asia Region and receives technical support for cancer registration from the International Agency for Research on Cancer (IARC), and the IARC Regional Hub based at the Tata Memorial Centre in Mumbai, India.

For data management of cancer registry records, Sri Lanka National Cancer Registry (SLNCR) uses the open-source registry software tool, CanReg5, as developed by IARC. The SLNCR has received data from 25 centres located across the country. Inputted data from the respective centres was then exported from various CanReg5 systems to the main centre in Colombo. As the import to the central CanReg5 system held in the capital is manual, the records were manually modified to avoid any duplicate entries, and the quality of data was compromised. To overcome this issue, a new software tool, Rupantaran, has been created and developed by IARC Regional Hub, Mumbai to help merge the records from different centres. Rupantaran was tested and implemented successfully at the SLNCR with 47,402 merged records. The Rupantaran software has proven beneficial in maintaining the quality of cancer registry data by avoiding manual errors, thus enabling rapid analysis and dissemination, a limiting factor previously.

## Background

A key area of focus of the International Agency for Research on Cancer (IARC) from the time of its inception has been to strengthen cancer registries in low and middle-income countries (LMICs). Cancer data has received greater attention for its role in cancer control and overall health system planning, producing increased demand for technical support. The Global Initiative for Cancer Registry Development (GICR, http://gicr.iarc.fr) was established in 2012 to markedly increase the coverage, quality and use of data from population-based cancer registries (PBCRs) in LMICs. Coordinated by IARC, the GICR brings together major international and national agencies committed to working collaboratively to improve cancer surveillance worldwide. Six IARC Regional Hubs serve as regional reference centres to coordinate GICR activities to expand assistance in cancer surveillance to countries using a coordinated approach based on local knowledge and expertise.

There are specific challenges in running PBCR in LMICs, including poor medical records, lack of cooperation from data providers, poor death registration systems, lack of trained registry staff, and difficulties in data entry and management [1]. In many LMICs, concerned ministries are often not in a position to provide the needed technical support and resources to cancer registries, including the provision of software for data management and statistical analysis.

To overcome the specific challenges in South and South-East Asia, IARC partnered with the Tata Memorial Centre (TMC) to establish an IARC Regional Hub in Mumbai in 2012 [3], with a remit to provide technical support for cancer registration to countries within the region and serve as the first point of contact to countries [4]. The Hub supports the operation of CanReg5 software as the data management software used by many PBCRs and provides technical assistance in data analysis and report writing to PBCRs where it is needed. The CanReg5 software is free, open-source and widely used by PBCR to input, manage, quality assure and analyse the data of cancer patients [2]. The CanReg5 software also has features to validate the entry of the data which helps maintain the quality of the same.

The Sri Lanka National Cancer Registry (SLNCR) regularly contacts the IARC Regional Hub, Mumbai for technical assistance and has installed the CanReg5 software in 25 centres across Sri Lanka to collect and manage the data of cancer patients. Data from these centres are regularly submitted to the central office in Colombo where these data are merged into the central CanReg5 database [5]. In CanReg5, new patient, tumour and source Identification (ID) are assigned for each entry. However, these new IDs are assigned sequentially and are used by CanReg5 for identifying duplicates in case consolidation between and within files. Thus, the same ID will appear for different patients in more than one centre. This results in incorrect data merging and has led to data-related problems which have impacted the functions of the SLNCR in recent years. The data cleaning process required significant time and effort, with merging the database a manual process and a major challenge to the registry staff.

The implementation of a virtual private network (VPN) can be a practical solution in dealing with data merging problems. A VPN extends a private network across a public network and enables users to send and receive data across shared or public networks, as if their computing devices were directly connected to the private network [6]. CanReg5’s client-server architecture feature can perform data entry simultaneously from different CanReg5 systems (client) so that the data get saved in the main CanReg5 system (server) in a centralized database [7]. If the VPN solution is implemented and used with the CanReg5 client-server architecture feature, the data merging problem can be resolved as data merging is no longer needed given the data has been saved centrally from the various centres. This solution could be replicated by other registries in the region where multiple centres are established.

However, implementing VPN has some limitations. It is not cost-effective. It is difficult to obtain funding from the Ministry of Health due to their prioritization of other public health programmes. Implementing a VPN network may result in administrative issues from different centres/hospitals, resulting from a lack of IT personnel and resources. Therefore, implementing a VPN in most LMICs is not likely to be feasible. There has been no means to merge exported data from CanReg5 software to another system that renumbers IDs to avoid duplication whilst maintaining the series of the record. The manual methods that have been developed proved complex, time-consuming and introduced errors. Following discussions between the SLNCR and the IARC Regional Hub, Mumbai, the new Rupantaran software was developed which allows automated merging of CanReg5 data collected from several centres. The main aim of the software is to merge CanReg5 data from various centres to the main centre. The software has been successfully installed at the central office of the SLNCR in Colombo. This article aims to provide technical details of the Rupantaran software and how its application resolved merging issues of CanReg5 data collected from different hospitals in Sri Lanka.

## Method

**Figure 1.**
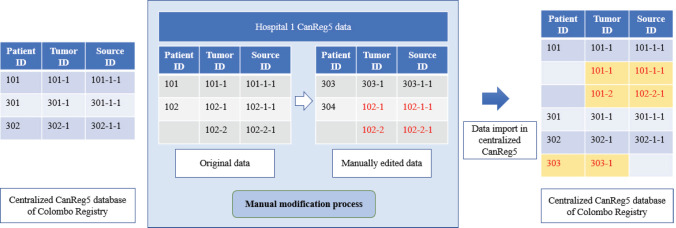
Manual modification of records for the process of data merging.

The word ‘Rupantaran’ is derived from the Marathi language, a language of Maharashtra state India, and signifies a ‘process of modification or conversion’. The CanReg5 software system is based on three tables for the entering details of the patient, tumour and source. The patient table saves patient’s basic and sociodemographic details, the tumour table saves tumour-related information and the source table saves information on the source. A patient can have multiple tumours, hence for a patient table record there can exist more than one tumour table record. Equally, a tumour can have multiple sources; hence, for a tumour table record, there can exist more than one source table record. A manual modification undertaken on such records can lead to the errors and is illustrated in Figure 1.

The Rupantaran software converts the IDs of patient, tumour and source tables as per the required numbering series; it modifies the data present in the following columns.

- Patient table columns
  - REGNO (Primary key)
  - PATIENTRECORDID
- Tumour table columns
  - TUMOURID (Primary key)
  - PATIENTRECORDIDTUMOURTABLE (Foreign key)
  - PATIENTIDTUMOURTABLE (Foreign key)
- Source table columns
  - SOURCERECORDID (Primary key)
  - TUMOURIDSOURCETABLE (Foreign key)

**Figure 2.**
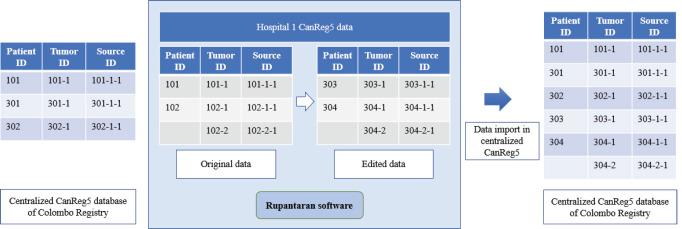
Modification of records through Rupantaran software for the process of data merging.

**Figure 3.**
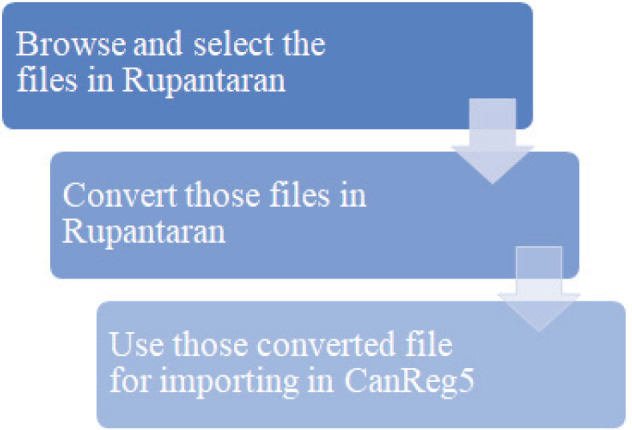
The steps to merge the CanReg5 data.

The modifications done through Rupantaran are shown in Figure 2. The column names in the CanReg5 software can vary for different registries. The steps to merge the CanReg5 data received from different centres to the main centre CanReg5 software using Rupantaran are illustrated in Figure 3. The steps to be followed are provided in Table 1.

During the conversion process, Rupantaran software takes the last unique ID of the CanReg5 software as input and later selects a patient record one at a time from the patient table selected by the user. For the selected patient record, the corresponding tumour record is searched in the tumour table. If the corresponding tumour record is found, then the corresponding source record is searched in the source table. When Rupantaran locates the tumour and source records for a given patient ID, a unique ID for each record is created. These unique IDs are the updated ID sequences. After the unique ID conversion of the selected patient record, the updated patient, tumour and source table data are saved in a new table. When the data conversion process is completed, the new tables which contain converted data are exported as tab-separated value (TSV) files. This results in the creation of new patient, tumour and source files. The name of those files has a format as (Table Name) _year (yyyy) -month (MM) -day (dd) hour (hh) _minutes (mm) _seconds (ss), e.g., ‘Patient_2022-10-15 17_32_18’.

Initially, the software was tested by the technical team at the IARC Regional Hub, Mumbai with minor errors observed and modified accordingly. The installation process and its application were demonstrated to the Sri Lanka Team. Following a period of testing by the SLNCR, the performance of the software was considered acceptable to address the issue of duplicate IDs. Subsequently, the software has been installed to merge the various cancer patient data files from several centres to the main database. The technical details and installation procedure are available as video clips online [8].

## Results

The Rupantaran software was introduced in SLNCR on 16th September 2021. The SLNCR has successfully merged 47,402 records using Rupantaran received from different hospitals. Moreover, as Rupantaran software creates a unique ID for each case, there is a complete elimination of concern of duplicates at the outset. The hospitals along with the number of records received centrally are provided in Table 2. The differences between the manual method and the use of Rupantaran software in merging and uploading the data are described in Table 3. Earlier 2 weeks were required for the data merging process along with four human resources. The same amount of data was merged in approximately 1 hour using Rupantaran software. Due to the software human hours, time was saved and the process was fast and more accurate. The time required for merging data to the central database has been reduced significantly, while errors introduced in merging the registry data manually have been eliminated.

## Discussion

As part of the GICR, the IARC Regional Hub in Mumbai is playing an important role in capacity building as well as providing technical solutions to PBCR. These services are freely available. The Hub has conducted 47 courses including in person as well as virtually and trained 1,190 participants across the South East Asia Regional Office (SEARO) region. The World Health Organization (WHO SEARO), New Delhi, India, is providing financial support to the Mumbai Hub to provide technical assistance to registries from countries within the SEARO region via a formal agreement between WHO SEARO and TMC. The cancer registries from Nepal, Sri Lanka, Myanmar, Timor Leste, Bhutan, Indonesia, Bangladesh, Thailand, Korea DPR, and Maldives are encouraged to contact the IARC Regional Hub, Mumbai where assistance is required.

The SLNCR staff has been trained by the IARC Regional Hub, Mumbai, in CanReg5 as well as the merging of data via the Rupantaran software. The registry team has started working effectively on the merging of the database and are in the process of analysing the data for publication in a timely report to following previous publications 2012–2019 available on the website [9]. The Rupantaran software complements CanReg5 by allowing the merging of data received from different sources and can also be used in office environments where local area network connectivity is not established.

Registries can have different lengths of unique ID for patient, tumour and source tables within CanReg5, and hence, Rupantaran needs to be specifically designed for a cancer registry with this in mind. The software can be updated as per specific requirements by the particular registries and the update is free of cost. The cancer registries from South-East Asia which are interested in implementing Rupantaran software should write an email to IARC Regional Hub, Mumbai (email id: budukham@tmc.gov.in and atul.budukh@gmail.com). The software is freely available and technical support will be provided by the Hub. The Rupantaran software works only on the Windows operating system.

During the CanReg5 virtual workshop organized by the IARC Regional Hub, Mumbai; Rupantaran was demonstrated to PBCR in Nepal, Bhutan, Afghanistan, and Timor Leste. As of now, these countries have their registry at one centre only. Later when these countries expand their registries, the option of using Rupantaran software should be considered for efficient and valid data merging. The software can be made available to the centres.

## Conclusion

The Rupantaran software has proven beneficial in maintaining the quality of cancer registry data by ensuring manual errors are avoided and, hence, enabling the SLNCR registry to work efficiently and progress with data collection, analysis and dissemination. Countries from the South-East Asian region are encouraged to contact the IARC Regional Hub in Mumbai for technical assistance in CanReg5 software, cancer registry training, and in collaboration in developing registry reports and scientific publications based on the collected data.

## List of abbreviations

IARC: International Agency for Research on Cancer; ID: Identification; LMIC: Low- and middle-income country; PBCR: Population-based cancer registry; SEARO: South-East Asian Regional Office; SLNCR: Sri Lanka National Cancer Registry; TMC: Tata Memorial Centre; TSV: Tab-separated values; VPN: Virtual private network; WHO: World Health Organisation.

## Conflicts of interest

The author(s) declare that they have no conflict of interest.

## Authors’ contributions

**Pratik Sawant:** Developed the software, provided technical guidance in data merging procedure, writing the manuscript.

**Suraj Perera:** Provided all technical input for the software, assisted in writing the manuscript, quality control.

**K.G. Nirmala Jayanthi:** Provided all technical input for the software, assisted in writing the manuscript, quality control.

**A. Ziyad:** Provided all technical input for the software, assisted in writing the manuscript, quality control.

**Sushama Saoba:** Assisted in developing the software, assisted in drafting the manuscript.

**Morten Ervik:** Quality control and assisting in writing the draft. 

**Leslie Mery:** Quality control and assisting in writing the draft.

**Freddie Bray:** Assisted in writing the manuscript.

**Rajesh Dikshit:** Assisted in writing the manuscript.

**Atul Budukh:** Supervision, conceptualisation, writing the manuscript, coordinating between the India and Sri Lanka team.

## Figures and Tables

**Table 1.** Steps needed to merge the CanReg5 data received from different centres to the centralized CanReg5 database using Rupantaran.

Steps	Action required
1	Receive the CanReg5 data tables – patient, tumour and source from each centre in TSV format.
2	In Rupantaran, browse and select the received patient, tumour and source TSV files.
3	Click on the convert files button. The Rupantaran software will ask you to enter a unique ID.
4	Enter the last unique ID from the centralized CanReg5 database and click start conversion.
5	Rupantaran will convert the selected files with the required unique ID sequence and will export those files in TSV format
6	Import those exported files into the main CanReg5 software.

**Table 2.** Number of cancer records received from Sri Lankan hospitals to the SLNCR for merging into one centralized CanReg5 database using the Rupantaran software.

Sr. No.	Name of the hospitals	Total number of records
1	District General Hospital, Ampara	90
2	Teaching Hospital, Anuradhapura	1,388
3	District General Hospital, Avissawella	247
4	Provincial General Hospital, Badulla	2,472
5	Teaching Hospital, Batticaloa	2,045
6	District General Hospital, Chilaw	256
7	District General Hospital, Gampaha	637
8	District General Hospital, Hambanthota	1,320
9	Teaching Hospital, Jaffna (Base Hospital – Tellipalai)	1,129
10	Teaching Hospital, Kalutara	Directly entered into the central database
11	National Hospital, Kandy	4,763
12	Teaching Hospital, Karapitiya	2,041
13	District General Hospital, Kegalle	320
14	Teaching Hospital, Kurunegala	1,524
15	District General Hospital, Matale	Data will be available in the next phase
16	District General Hospital, Matara	Data will be available in the next phase
17	District General Hospital, Monaragala	387
18	District General Hospital, Nuwaraeliya	354
19	District General Hospital, Polonnaruwa	140
20	Teaching Hospital, Ragama	189
21	Teaching Hospital, Rathnapura	2,451
22	District General Hospital, Trincomalee	265
23	District General Hospital, Vavuniya	264
24	Pathology laboratory data received in Excel sheet format^a^	7,332
25	Apeksha Hospital (National Cancer Institute)	17,788
Total		47,402

a Pathology laboratory data received through Excel sheet format – Coded according to ICD O manually. Prepared columns according to the Excel sheet format of CanReg 5 and uploaded them into an empty CanReg 5 database. From that database, tumour, patient and source TSV formats were generated to be uploaded via Rupantaran as described above

**Table 3.** Differences between manual methods and the use of Rupantaran software in data merging and uploading.

Manual method	Rupantaran software
More person-time required.	Approximately 1 hour required
Manual merging using Excel	The process is automatic using dedicated software
Manual errors in patient, tumour and source IDs	No errors in patient, tumour and source IDs
Data inconsistency high	No data inconsistencies
Delay in data analysis as merging of data is tedious and takes time	No delay in data analysis as the merging of data is accurate and rapid

## References

[1] Manual for Cancer Registry Personnel (1995). International Agency for Research on Cancer: Technical Report No. 10.

[2] Ervik M, Cooke A, Ferlay J (2008). CanReg5 Open Source Software for Cancer Registries.

[3] International Agency for Research on Cancer International Cancer Community Welcomes Global Initiative for Cancer Registry Development in Low- and Middle-Income Countries.

[4] Global Initiative for Cancer Registry Development South East and South-Eastern Asia Hub [Internet].

[5] National Cancer Control Program, Ministry of Health, and Nutrition & Indigenous Medicine (2019). Standard Operating Procedures for Cancer Registration in Sri Lanka.

[6] What is a VPN? – virtual private network [Internet]. https://www.cisco.com/c/en_in/products/security/vpn-endpoint-security-clients/what-is-vpn.html.

[7] Ervik M (2023). CanReg5 – Instructions (Version February 2023).

[8] Rupantaran software- installation and working [Internet]. https://youtu.be/TDiI48u1mxA.

[9] National Cancer Control Program, Ministry of Health Nutrition & Indigenous Medicine, Sri Lanka [Internet] https://www.nccp.health.gov.lk/en.

